# Children with atopic eczema experiencing increased disease severity in the pollen season more often have hay fever at a young age and a dark skin type

**DOI:** 10.1111/1346-8138.15750

**Published:** 2021-01-06

**Authors:** Angela Leigh‐Ann Bosma, Wouter Ouwerkerk, Maritza Albertina Middelkamp‐Hup

**Affiliations:** ^1^ Amsterdam UMC, location Academic Medical Center Department of Dermatology Amsterdam Public Health, Infection and Immunity University of Amsterdam Amsterdam The Netherlands; ^2^ National Heart Center Singapore Singapore

**Keywords:** atopic eczema/dermatitis, hay fever, phenotype, pollen season, skin type

## Abstract

Children with atopic eczema are known to experience seasonal variations in disease severity, with winter being the season in which severity generally increases. There is a lack of knowledge about the subgroup of children that experiences increased severity in spring and summer months. We aimed to investigate which phenotype characteristics best describe children flaring in the pollen season. A retrospective database analysis was conducted, including 110 children with difficult‐to‐treat atopic eczema aged 0–17 years. Relevant outcome parameters were extracted from medical records. In our population, 36% (n = 40/110) of children reported flares of atopic eczema in the pollen season. These children were more often sensitized to one or more types of pollen (73% [n = 29/40] vs. 28% [n = 10/36], *p* < 0.0001) and had more patient‐reported hay fever (70% [n = 28/40] vs. 19% [n = 7/36], *p* < 0.0001), compared with children who do not flare in the pollen season. Moreover, children flaring in the pollen season more often had a dark skin type (78% [n = 31/40] vs. 44% [n = 16/36], *p* = 0.003). Based on stepwise multivariable analyses, children flaring in the pollen season were characterized by the combination of younger age, hay fever, and dark skin type (C‐statistic: 0.86). In conclusion, patient‐reported flares in spring and summer are experienced by one‐third of children with difficult‐to‐treat atopic eczema. This phenotype can be characterized as young children having hay fever and a dark skin type and can be identified based on clinical parameters alone without the need to perform immunoglobulin E blood testing or skin prick tests.

**Abbreviations**
AE: atopic eczema
AIC: Akaike information criterion
CI: confidence interval
IgE: immunoglobulin E
OR: odds ratio

## 1 INTRODUCTION

Atopic eczema is one of the most common skin disorders, affecting up to 20% of children in the general population.^1^ The disorder is characterized by pruritic inflammation of the skin and can have a major impact on the quality of life of patients and their families. AE is considered both an immunological and skin barrier disorder.^2^, ^3^ Children with AE often have allergic comorbidities. Presence of eczema in the first 2 years of life is a risk factor for both allergic rhinoconjunctivitis and asthma.^4^ Patients with AE are significantly more sensitized to aeroallergens than children without AE. Pollen prove to be one of the most common relevant aeroallergens in these patients.^5^, ^6^

In clinical practice, patients with AE experience seasonal variations in disease severity. Besnier^7^ described AE as a familial pruritic skin disease starting at a young age, followed by a chronic fluctuating course with seasonal variations, that often occurs in combination with allergic rhinoconjunctivitis and asthma. In general, AE symptoms are worse in winter, due to potential dehydration of the skin by exposure to low humidity in the environment (e.g., cold weather and central heating).^8^ However, a subgroup of patients experiences disease flares in spring and summer months.

We hypothesize that there are two possible subtypes of AE: one that is worsened in spring and summer, and the other that is worsened in winter. The objective of this study was to characterize the phenotype of children with AE who experience flares during spring and summer months. First, we investigated whether sensitization to pollen and/or hay fever are associated with flaring in spring and summer (the pollen season). Next, we investigated what other phenotype characteristics, in particular having asthma and the patient’s skin type, are distinguishing for this subgroup.

## 2 METHODS

### 2.1 Study design

A retrospective database analysis was performed at the specialized day‐care treatment unit of the Department of Dermatology of the Amsterdam UMC in the Netherlands. Predetermined outcome parameters were extracted from a standardized intake questionnaire, including the demographic characteristics sex, date of birth, and Fitzpatrick skin type. During the intake, children and their caregivers were asked about seasonal variations in AE symptoms, by inquiring about an increase of their symptoms in the pollen season. The parameters hay fever and asthma were defined as patient‐reported current or history of hay fever and asthma. Results from total IgE levels, and from radioallergosorbent tests and skin prick tests, were collected to detect allergen‐specific IgE levels against grass, tree, and herb pollen. Sensitization was considered present in case of a positive prick skin test or more than 3.50 kU/L allergen‐specific IgE antibodies against pollen in serum. In case no information on either skin prick testing or IgE levels was present, this data was considered missing. Light skin types were defined as skin types 1, 2, and 3, and skin types 4, 5, and 6 as dark skin types. Obtaining ethical approval was not necessary according to the Medical Ethics Committee of the Amsterdam UMC (W20_308 #20.343). The study was carried out in accordance with the provisions of the Declaration of Helsinki.

### 2.2 Patient population

The cohort consisted of children with difficult‐to‐treat AE (≤17 years) referred to our specialized day‐care treatment unit for AE because of unresponsiveness to conventional outpatient treatments such as topical and/or systemic therapy from January 2011 till October 2015. All of the included children were considered to have physician‐assessed AE, diagnosed by a dermatologist.

### 2.3 Statistical analyses

Descriptive differences between patient characteristics were compared using the χ^2^‐test, Mann–Whitney *U*‐test or unpaired *t*‐test where appropriate.

In addition, to find the combination of variables to describe children with AE flaring in the pollen season, we performed stepwise logistic regression analyses, by AIC, using 500 bootstrap samples in five imputed datasets.^9^ We considered the following variables to be selected in our model: age, sex, dark versus light skin type, asthma, allergic rhinoconjunctivitis, and sensitization to grass, tree, and herb pollen. Missing values were imputed using an expectation–maximization algorithm with bootstrapping approach.^10^ We developed one clinical model where the variables based on pollen sensitization were excluded, as blood or skin prick tests are not always performed in daily practice, especially not in younger children. To investigate the additional value of the variables based on pollen sensitization, we included these variables in an extended model as a sensitivity analysis.

Statistical analysis of the data was performed using the software program SPSS Statistics 23 (IBM, Armonk, NY, USA) and R: Language and Environment for Statistical Computing version 3.6.1 (R Foundation for Statistical Computing, Vienna, Austria). Results were considered statistically significant at *p* < 0.05 for all analyses.

## 3 RESULTS

This study included 110 children, 58 boys and 52 girls, with a mean age of 7.7 years (±5.1 SD; Table 1). The majority of children had a dark skin type (65%, n = 71). Flaring of AE in the pollen season was experienced in 36% (n = 40) of the children, and 33% (n = 36) did not experience pollen season‐related flares. For 17% (n = 19), the relation between the pollen season and AE was unclear or unknown, and 14% (n = 15) of the children experienced their first flare of AE.

**Table 1:** Patient characteristics

Patient characteristics (n = 110)	
Sex, n (%)	
Male	58 (53%)
Female	52 (47%)
Age, mean ± SD (range)	7.7 ± 5.1 years (0–17)
Skin type, n (%)	
I	1 (1%)
II	12 (11%)
III	24 (22%)
IV	29 (26%)
V	23 (21%)
VI	19 (17%)
Unknown	2 (2%)
Skin type, median	4
Pollen season‐related flares	
Yes	40 (36%)
No	36 (33%)
Unknown	19 (17%)
First exacerbation	15 (14%)
Total IgE level, median (IQR)^a^	1513.00 kU/L (415.00–4500.00)

IgE, immunoglobulin E; IQR, interquartile range.

^a^ n = 51

Children with pollen season‐related flares were found to be more sensitized to one or more types of pollen compared with children who do not flare in the pollen season (73% [n = 29/40] vs. 28% [n = 10/36], *p* < 0.0001). This association was also seen separately for sensitization to tree pollen (50% [n = 20/40] vs. 22% [n = 8/36], *p* = 0.004) and grass pollen (60% [n = 24/40] vs. 28% [n = 10/36], *p* = 0.003), but not herb pollen (Table 2). We also found that children with pollen season‐related flares had more patient‐reported hay fever (70% [n = 28/40] vs. 19% [n = 7/36], *p* < 0.0001), compared with children without pollen season‐related flares. There was a trend observed for an increased prevalence of asthma in the group experiencing pollen season‐related flares (48% [n = 19/40] vs. 25% [n = 9/36], *p* = 0.05). Although higher median total IgE values were found in patients flaring in the pollen season, no statistical difference was found (2087.0 vs. 1404.5, *p* = 0.26).

**Table 2:** Patient characteristics regarding pollen season‐related flares

Patient characteristics regarding pollen season‐related flares (n = 76)			
	Patients with pollen season‐related flares (n = 40)	Patients without pollen season‐related flares (n = 36)	*p*
Sex (% of total, n: male vs. female)	50% vs. 50% (20 vs. 20)	47% vs. 53% (17 vs. 19)	0.81
Age (mean ± SD, range)	9.0 ± 4.6 years (2–17 years)	9.0 ± 4.8 years (2–17 years)	0.96
Skin type (% of total, n)	Light: 23% (9) Dark: 78% (31)	Light: 56% (20) Dark: 44% (16)	**0.003**
Sensitization (% of total, n)^a^	Tree pollen: 50% (20) Grass pollen: 60% (24) Herb pollen: 15% (6) Combined (≥1 types of pollen)^b^: 73% (29)	Tree pollen: 22% (8) Grass pollen: 28% (10) Herb pollen: 8% (3) Combined (≥1 types of pollen)^c^: 28% (10)	**0.004** **0.003** 0.52 **<0.0001**
Hay fever^d^ (% of total, n)	70% (28)	19% (7)	**<0.0001**
Asthma^e^ (% of total, n)	48% (19)	25% (9)	0.05
Total IgE level^f^ (median [IQR])	2087.0 (556.5–5320.0) kU/L	1404.5 (362.8–3265.3) kU/L	0.26

IgE, immunoglobulin E; IQR, interquartile range.

Significant results are presented in bold face.

^a^ Missing data: tree pollen, n = 23; grass pollen, n = 19; herb pollen, n = 27; and combined pollen, n = 21.

^b^ Sensitization to one or more types of pollen.

^c^ Missing data: n = 8.

^d^ Missing data: n = 3.

^e^ Missing data: n = 39

Interestingly, children with a dark skin type more often experienced pollen season‐related flares of AE (78%, n = 31/40), while in the group without pollen season‐related flares only 44% (n = 16/36) of the children had a dark skin type (*p* = 0.003). When we stratified our population based on skin type (Table 3), sensitization to one or more types of pollen was more prevalent in dark skin types in comparison with light skin types (52% [n = 37/71] vs. 46% [n = 17/37], *p* = 0.04). There were no significant differences found for sensitization to tree, grass, and herb pollen separately (*p* = 0.85, 0.34, and 0.69, respectively). Children with dark skin types more often had hay fever compared with children with light skin types (41% [n = 29/71] vs. 30% [n = 11/37]), although we did not find a significant difference (*p* = 0.15). No statistical difference was found for median total IgE values between patients with light and dark skin types (1513.0 vs. 1765.0, *p* = 0.27).

**Table 3:** Patient characteristics regarding skin type

Patient characteristics regarding skin type (n = 108)			
	Light skin type^a^ (n = 37)	Dark skin type^b^ (n = 71)	*p*
Sex (% of total, n: male vs. female)	65% vs. 35% (24 vs. 13)	47% vs. 54% (33 vs. 38)	0.08
Age (mean ± SD, range)	7.9 ± 5.2 (0–17 years)	7.8 ± 5.1 (0–17 years)	0.97
Sensitization^c^ (% of total, n)	Tree: 43% (16) Grass: 43% (16) Herb: 16% (6) Combined (≥1 types of pollen)^d^: 46% (17)	Tree: 35% (25) Grass: 42% (30) Herb: 14% (10) Combined (≥1 types of pollen)^c^: 52% (37)	0.85 0.34 0.69 **0.04**
Pollen season‐related flares (% of total, n) Yes No	24% (9) Skin type 1: ‐ Skin type 2: 8% (3) Skin type 3: 16% (6) 54.0% (20) Skin type 1: ‐ Skin type 2: 16% (6) Skin type 3: 38% (14)	44% (31) Skin type 4: 16% (11) Skin type 5: 14% (10) Skin type 6: 14% (10) 22.5% (16) Skin type 4: 11% (8) Skin type 5: 7% (5) Skin type 6: 4% (3)	**0.003**
Hay fever^e^ (% of total, n)	30% (11)	41% (29)	0.15
Asthma^f^ (% of total, n)	32% (12)	31% (22)	0.76
Total IgE level^g^ (median [IQR])	1513.0 (386.0–2372.0) kU/L	1765.0 (444.8–5798.8) kU/L	0.27

IgE, immunoglobulin E; IQR, interquartile range.

Significant results are presented in bold face.

^a^ Light skin types were defined as skin type 1, 2, and 3.

^b^ Dark skin types were defined as skin type 4, 5, and 6.

^c^ Missing data: tree pollen, n = 33; grass pollen, n = 29; herb pollen, n = 41; and combined pollen, n = 33.

^d^ Sensitization to one or more types of pollen.

^e^ Missing data: n = 18.

^f^ Missing data: n = 12.

^g^ Missing data: n = 57

**FIGURE 1:**
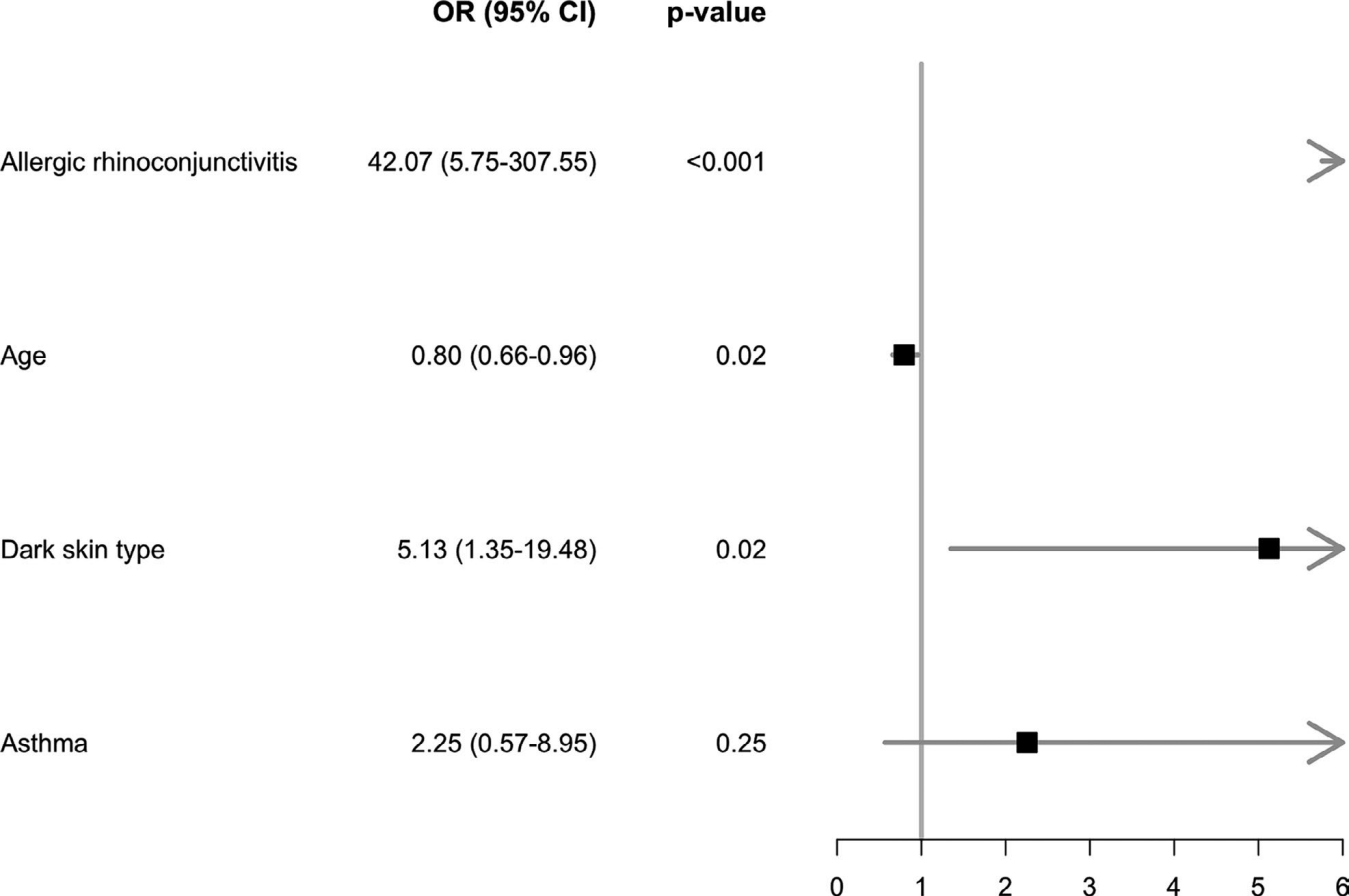
Forest plot main analysis. The figure displays the selection of the most important characteristics based on our stepwise logistic regression model. The following variables were included: age, sex, dark versus light skin type, asthma, and allergic rhinoconjunctivitis. CI, confidence interval; OR, odds ratio

Multivariable stepwise regression analysis (Figure 1) showed a combination of hay fever (OR = 42.07; 95% CI, 5.75–307.55; *p* < 0.001), dark skin type (OR = 5.13; 95% CI, 1.35–19.48; *p* = 0.02) and age (OR = 0.80; 95% CI, 0.66–0.96; *p* = 0.02) to be the most important characteristics associated with pollen season‐related flares. Asthma was also seen more often in children with pollen season‐related flares (OR = 2.25; 95% CI, 0.57–8.95; *p* = 0.25). Bootstrapped optimism corrected C‐statistic of the model without the variables based on pollen sensitization was 0.86, and 0.88 for the model including these variables (Figure S1). Both models were able to identify children with pollen season‐related flares, showing that the addition of information on pollen sensitization did not significantly improve the model (*p* = 0.93).

## 4 DISCUSSION

We showed that a subgroup (36%) of children with difficult‐to‐treat AE experiences pollen season‐related flares. Patients flaring in the pollen season more often have sensitization to pollen, hay fever, and a dark skin type. In general, 60% or more of inhalant allergen sensitizations are thought to be clinically relevant, with the highest clinical relevance seen for sensitization to grass pollen.^11^ In our population, the most common sensitization was against grass pollen, and children with pollen season‐related flares of AE more often seemed to have hay fever as a clinically relevant manifestation of immunological sensitization.

Seasonal flares of AE have been described in the literature, but research on its associated phenotype characteristics is sparse. A study performed in Japan investigated 682 AE patients, of whom 66% reported seasonal worsening of their symptoms. The proportion of patients with flares in spring and summer was 25% and 19%, respectively, with 11% and 36% experiencing flares during autumn and winter.^12^ A German study identified two patterns of seasonal flares among children with AE, with 54% of their subjects experiencing symptoms mainly in winter and the remaining 46% mainly in summer. In the latter group, symptoms were worse with high pollen exposure, particularly in patients sensitized to pollen.^13^ A study measuring skin symptoms in Korean children with AE demonstrated worse symptoms in spring, autumn, and winter relative to summer, with April as the worst month.^14^

We found that the best combination of characteristics associated with flaring in the pollen season are younger age, hay fever, and dark skin type, indicating that younger children with hay fever and a dark skin type are at increased risk of pollen season‐related flares. Children with dark skin who already have developed hay fever at a young age probably represent an immunologically different subgroup within the AE population. Our model showed that asthma may also be an important characteristic, but larger studies are needed to confirm this association.

Out of all of the variables, hay fever was the most important with an OR of 42.07 in our main analysis. Our model selected the combination of the least variables that best describes the outcome. Our sensitivity analysis showed that adding pollen sensitization to the model did not increase the accuracy of predicting flaring in the pollen season. Based on this we can conclude that in clinical practice, children flaring in the pollen season can be identified based solely on clinical characteristics and therefore performing blood or skin prick tests is not necessary for this specific aim.

Our results showed that children with dark skin types more often experience flares of AE in the pollen season. We also found that children with dark skin types are more often sensitized to pollen and we believe this could be the explanation for flaring in the pollen season. In the literature, it has been demonstrated that AE is more prevalent in black and mixed race populations, with genetics a possible cause.^15^, ^16^ An increased prevalence of sensitization among dark skin types has also been demonstrated in other studies.^17^, ^18^ In addition to a potential genetic predisposition, racial disparities in sensitization and allergies may represent exposure to environmental factors, such as location of residence, socioeconomic status, and/or education.^18^, ^19^ We should be aware of the possibility that children with dark skin are more likely to flare during spring and summer months.

Being a retrospective study, our findings depend on pre‐existing and occasionally incomplete documentation in medical records, despite all efforts to record all data. Moreover, we based our findings on self‐reported increases of disease severity, rather than clinically observed flares measured with severity scores. The term “pollen season” is subject to some extent of interpretation by children and their caregivers. In addition, the pollen season is indirectly accompanied by exposure to other external factors that have the ability to influence the course of AE. For example, sweating and heat can cause pruritus.^20^, ^21^ An abnormal sweating response is observed in patients with AE.^20^, ^22^ Also, patients can have allergies for sunscreen ingredients.^23^

As for the therapeutic management of flares of AE in the pollen season, it is important to make these patients aware of their risk for flaring in the pollen season. Patients may benefit from avoiding pollen exposure, treatment with antihistamines, and treatment targeting improvement of the skin barrier function.^24^ Allergen‐specific immunotherapy is also suggested, but has not been demonstrated valuable yet.^25^ Treatment adherence impacts the utility of this option.^26^

In conclusion, our findings indicate that patient‐reported pollen season‐related flares of AE are present in approximately one‐third of children with difficult‐to‐treat AE. Sensitization to one or multiple types of pollen and current or history of hay fever occurs significantly more frequently in this subgroup. Furthermore, children with dark skin types more often experience flaring in the pollen season than children with light skin types. Sensitization to one or more types of pollen is more prevalent in dark skin types in comparison with light skin types. The phenotype of children with AE flares in the pollen season is best characterized by having hay fever, being of young age, and having a dark skin type.

## CONFLICT OF INTEREST

None declared.

## Supporting information


**Figure S1.** This figure displays the selection of the most important characteristics based on our stepwise logistic regression model. The following variables were included: age, gender, dark vs light skin type, asthma, allergic rhinoconjunctivitis, grass pollen sensitisation, tree pollen sensitisation, herb pollen sensitisation. CI, confidence interval; OR, odds ratio
