# Supplementary figures and images for: Children with atopic eczema experiencing increased disease severity in the pollen season more often have hay fever at a young age and a dark skin type
Source: J Dermatol. 2021 Jan 6;48(4):470–5. doi: 10.1111/1346-8138.15750 (PMC8048828; doi:10.1111/1346-8138.15750)

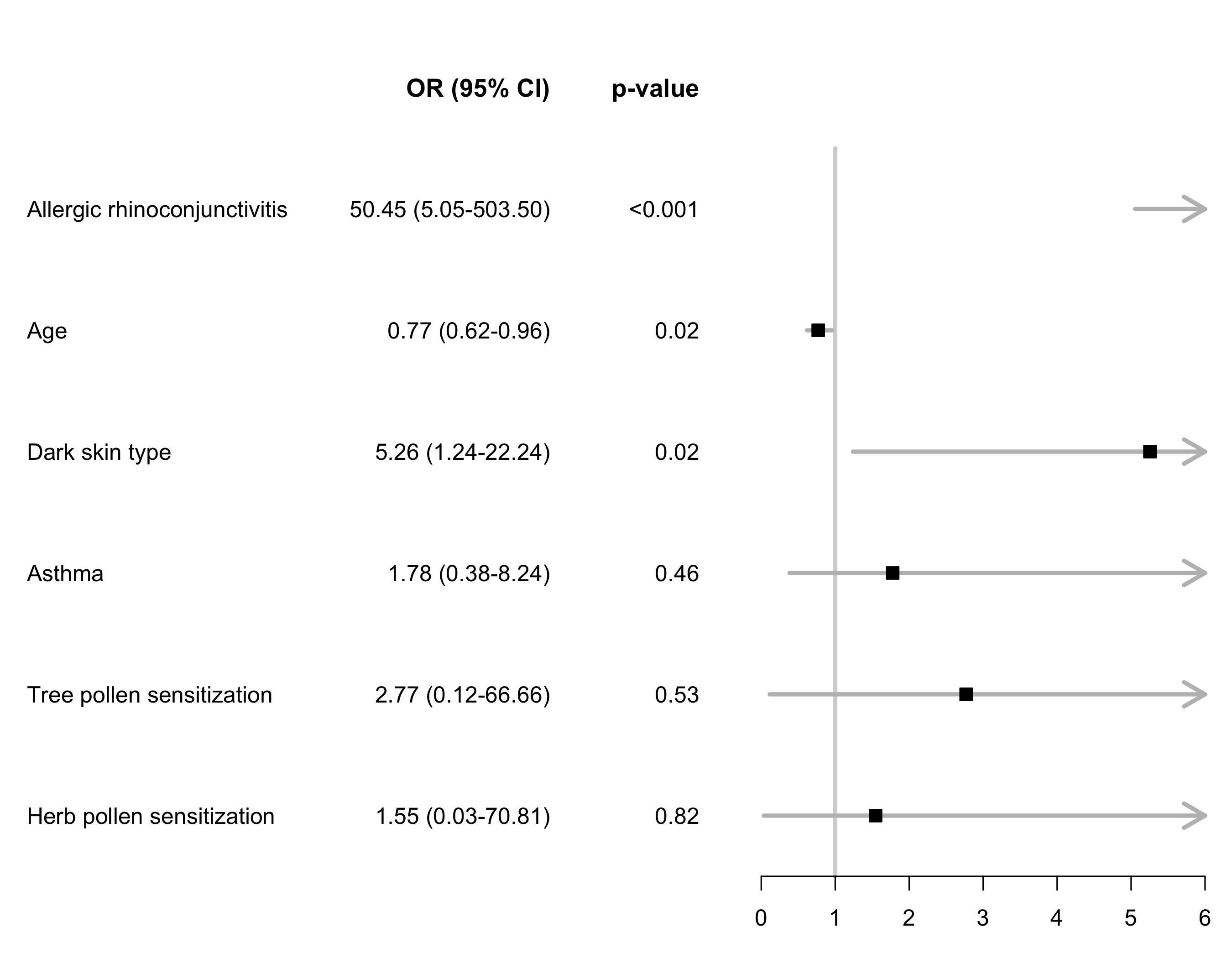

Supplement: Supplementary file 1 — Figure S1. This figure displays the selection of the most important characteristics based on our stepwise logistic regression model. The following variables were included: age, gender, dark vs light skin type, asthma, allergic rhinoconjunctivitis, grass pollen sensitisation, tree pollen sensitisation, herb pollen sensitisation. CI, confidence interval; OR, odds ratio [file JDE-48-470-s001.tiff]
